# DGIdb 2.0: mining clinically relevant drug–gene interactions

**DOI:** 10.1093/nar/gkv1165

**Published:** 2015-11-03

**Authors:** Alex H. Wagner, Adam C. Coffman, Benjamin J. Ainscough, Nicholas C. Spies, Zachary L. Skidmore, Katie M. Campbell, Kilannin Krysiak, Deng Pan, Joshua F. McMichael, James M. Eldred, Jason R. Walker, Richard K. Wilson, Elaine R. Mardis, Malachi Griffith, Obi L. Griffith

**Affiliations:** 1 McDonnell Genome Institute, Washington University School of Medicine, St. Louis, MO 63108, USA; 2 Siteman Cancer Center, Washington University School of Medicine, St. Louis, MO 63110, USA; 3 Department of Medicine, Washington University School of Medicine, St. Louis, MO 63110, USA; 4 Department of Genetics, Washington University School of Medicine, St. Louis, MO 63110, USA

## Abstract

The Drug–Gene Interaction Database (DGIdb, www.dgidb.org) is a web resource that consolidates disparate data sources describing drug–gene interactions and gene druggability. It provides an intuitive graphical user interface and a documented application programming interface (API) for querying these data. DGIdb was assembled through an extensive manual curation effort, reflecting the combined information of twenty-seven sources. For DGIdb 2.0, substantial updates have been made to increase content and improve its usefulness as a resource for mining clinically actionable drug targets. Specifically, nine new sources of drug–gene interactions have been added, including seven resources specifically focused on interactions linked to clinical trials. These additions have more than doubled the overall count of drug–gene interactions. The total number of druggable gene claims has also increased by 30%. Importantly, a majority of the unrestricted, publicly-accessible sources used in DGIdb are now automatically updated on a weekly basis, providing the most current information for these sources. Finally, a new web view and API have been developed to allow searching for interactions by drug identifiers to complement existing gene-based search functionality. With these updates, DGIdb represents a comprehensive and user friendly tool for mining the druggable genome for precision medicine hypothesis generation.

## INTRODUCTION

With the increasing availability and decreasing cost of molecular profiling methods, growing attention has been paid to the use of these technologies for characterizing the mechanisms of human disease at the cohort—and more recently, the individual—level (1–3). Improved understanding of such molecular characteristics has been demonstrated to impact the diagnostic, prognostic, and treatment decisions made by clinicians, yielding positive results, less toxicity and improved quality of life (4,5). However, finding well-annotated drug–gene interactions relevant to medical decision-making remains an ongoing challenge. Several resources exist that characterize both established and experimental drug–gene interactions but these resources vary widely in data structure and complexity. Many of these databases are missing useful features provided by DGIdb, such as tools for querying lists of terms (6,7), filtering based on interaction type (6–9) or gene class (7–9), and a RESTful API (7–10). Others lack a search interface entirely, and instead have only a browsing interface (8) or are available only as static supplementary documents published online (11–20).

DGIdb 1.0 (21) was first introduced as a novel resource to enable mining of multiple existing sources of drug–gene interactions and druggable gene categories. *drug–gene interactions* are the observed or inferred interaction between gene products (*targets*) and drug compounds (*ligands*) obtained by literature mining or by parsing publicly available databases. Many of these interactions include information on *interaction types* that specify how the drug interacts with the gene. The curation of DGIdb sources for interaction type data resulted in 33 distinct interaction type terms. In simple cases, these terms were harmonized where the interaction types were unquestionably equivalent (e.g. ‘inhibitor’ = ‘inhibitors’). However, in most cases we felt it best to preserve any subtleties of the interaction type according to the source. *Druggable gene categories* include genes that currently may not be targeted therapeutically but are potentially druggable according to their membership in gene categories associated with druggability (e.g. kinases) (21). These categories for the most part were defined by the original seminal paper by Hopkins and Groom (12) who first defined the ‘druggable genome’ as genes belonging to over 130 specific biological types (kinases, nuclear hormone receptors, etc.) that were thought to be druggable, and our curation of these types reduced that definition to 21 distinct types that were incorporated into DGIdb. Russ and Lampel (16) published an update of the ‘Druggable Genome’ concept but without breaking down genes into specific categories. The dGene resource (13) was the most recent update to the concept of a ‘Druggable Genome’ list. These lists, while widely cited and used, were not available in any conveniently usable form. Therefore, we created a generic ‘Druggable Genome’ category, comprised of the union of these three sources to act as a convenient label for these definitive lists. Other categories from other sources are more specific. The ‘Clinically Actionable’ category is a new concept with the DGIdb 2.0 release describing lists of genes that are actively being used in targeted clinical sequencing panels for precision medicine in cancer. The druggable gene categories of DGIdb are by no means mutually exclusive, and in fact often overlap extensively if not entirely (serine threonine kinases, for instance, are a subset of all kinases).

Populating DGIdb 1.0 was accomplished through an extensive curation effort. This process typically included manually extracting and reviewing records, converting them to represent the concepts stored in the DGIdb, and writing custom importers to load the data provided by each source. Thirteen distinct sources were curated into a broad resource that is simple to query and explore. Druggable gene categories could be browsed without any structured query, and the interface accepted most common gene nomenclatures as identifiers for searching drug–gene interactions. Data describing interactions or potentially druggable categories (referred to as interaction or category *claims*) from each of these sources were linked to corresponding drug and gene concepts. Each such source typically included important metadata characterizing aspects of the interaction, such as if a particular gene confers resistance or sensitivity to the drug.

The breadth, quality, and ease-of-access enabled by this resource is reflected in the substantial web traffic DGIdb receives, with ∼1500 unique users and ∼2300 sessions a month, with an average duration of ∼4.4 min/session. In addition, a number of other bioinformatics tools have used DGIdb in developing their own platforms including PANDA (22), iCAGES (icages.usc.edu), BioGPS (23), OmicsPipe (24), GEMINI (25), StationX (www.stationxinc.com) and IHLDB.rf (www.lungcancerdatabase.com), highlighting the broad usefulness of the DGIdb API. The first version of DGIdb was released in October 2013, and has since been cited in over 50 publications.

Despite its popularity, DGIdb 1.0 was limited in several ways. The gene-centric search interface allowed the user to find known drug interactions for a set of gene targets, but did not provide a straightforward way of querying known gene targets for a set of drugs. In addition, several popular resources describing the druggable genome and drug–gene interactions were missing from DGIdb. Finally, some of the resources parsed by DGIdb are continually updated, creating a need for repeated manual updates to keep the associated data current.

**Figure 1.**
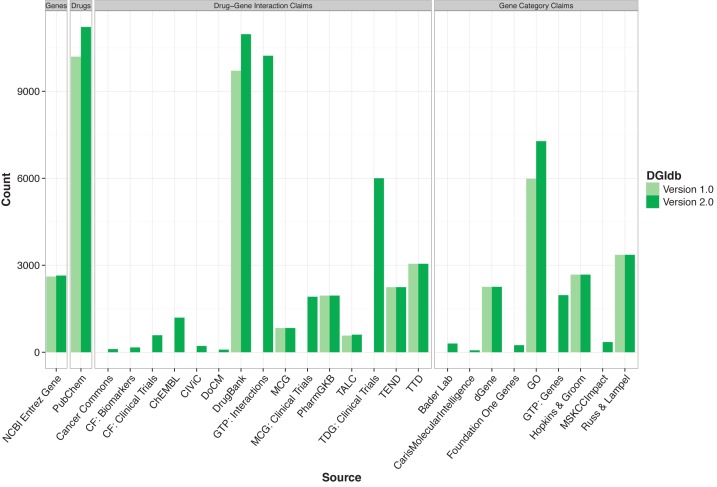
DGIdb 1.0 and 2.0 content by source. Here, the number of genes with interactions (first panel), drug claims (second panel), drug–gene interaction claims (third panel), and druggable gene categories (fourth panel) are contrasted between DGIdb 1.0 and 2.0. In total, there are currently 2,644 (33 new to DGIdb 2.0) genes with interactions, 11,215 (1,023 new) drug claims, 40,017 (21,624 new) drug–gene interaction claims, and 18,500 (4,224 new) gene category claims. Abbreviations: CF = Clearity Foundation, GTP = Guide To Pharmacology, MCG = My Cancer Genome, TALC = Targeted Agents in Lung Cancer, TTD = Therapeutic Target Database, TEND = Trends in the Exploration of Novel Drug targets, and GO = Gene Ontology MSKCC = Memorial Sloan Kettering Cancer Center.

With this update, we address these shortcomings. DGIdb 2.0 includes 14 new sources added since version 1.0 for a cumulative total of 27 sources describing drug–gene interactions and druggable gene categories (Tables 1 and 2). These include nine new sources describing 10,102 drug–gene interactions potentially relevant to clinical actionability (e.g. relevant clinical trials, etc.). With this focus, DGIdb is better positioned as a resource for drug–gene interaction hypothesis generation in precision medicine efforts. By reviewing drug–gene interactions that are clinically relevant, basic researchers and physician-scientists using DGIdb have a more useful reference for hypothesizing which treatments might provide benefits for individual patients. Of special note are the addition of drug–gene interactions from the Clinical Interpretations of Variants in Cancer (CIViC, www.civicdb.org) database and the Database of Curated Mutations (DoCM, www.docm.info), each of which are a curated source of clinically relevant cancer genes and their therapeutic interactions. DGIdb 2.0 also contains three new sources of potentially druggable gene category annotations, with 2,928 putatively druggable gene category assignments between them (Figure 1). These additions enhance the existing capacity of DGIdb as a tool for prioritizing development of new targeted therapies. Selected sources of gene, drug, interaction, and druggable categories are also now updated automatically, keeping DGIdb current as more information is gathered and deposited in the various sources aggregated by DGIdb. In addition to content updates, DGIdb 2.0 also supports a major new feature—searching interactions by drug identifier—which allows users to find drug–gene interactions by querying the database with drug identifiers in addition to gene identifiers.

**Table 1.** Description of drug–gene interaction sources in DGIdb 2.0

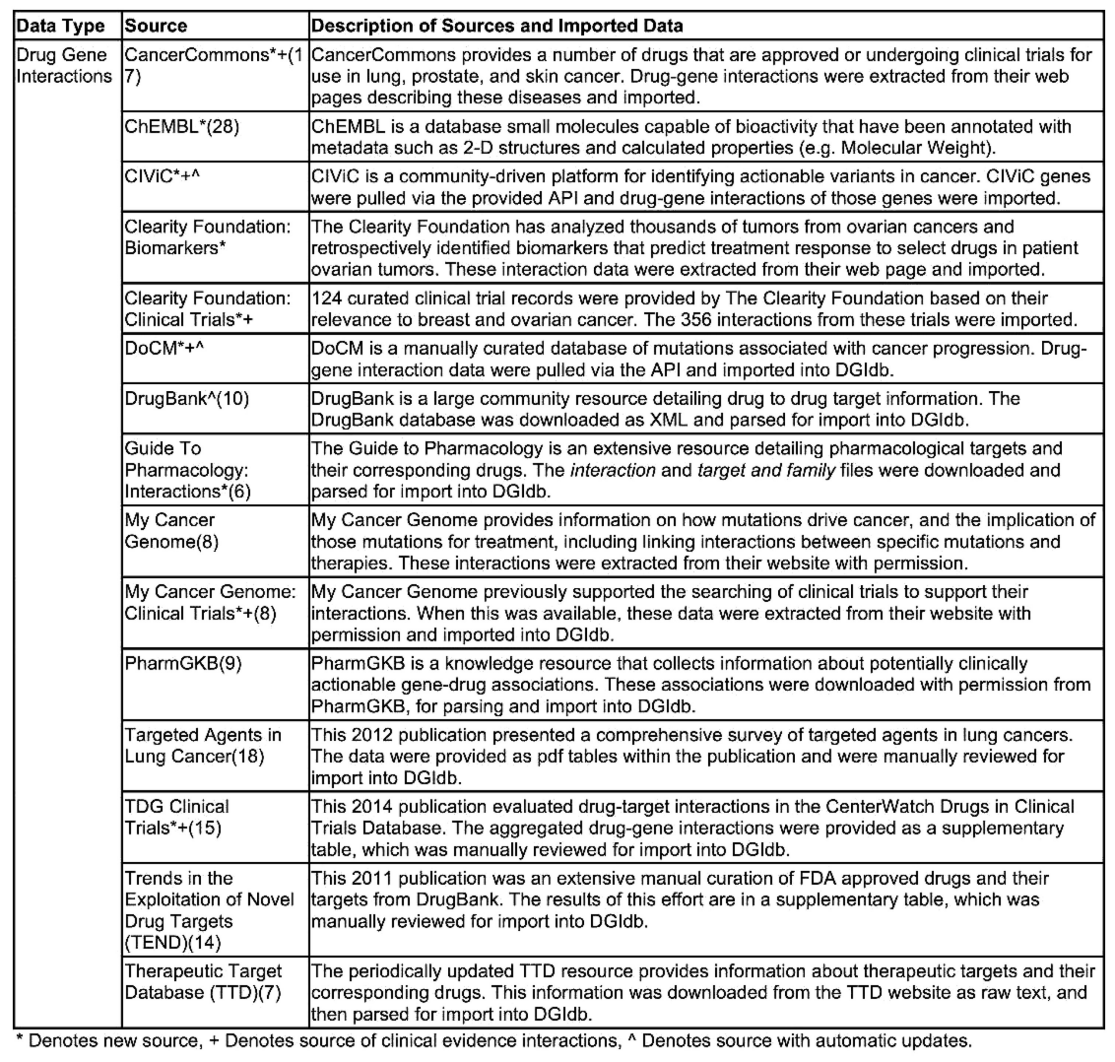		

**Table 2.** Description of drug, gene and druggable gene category sources in DGIdb 2.0

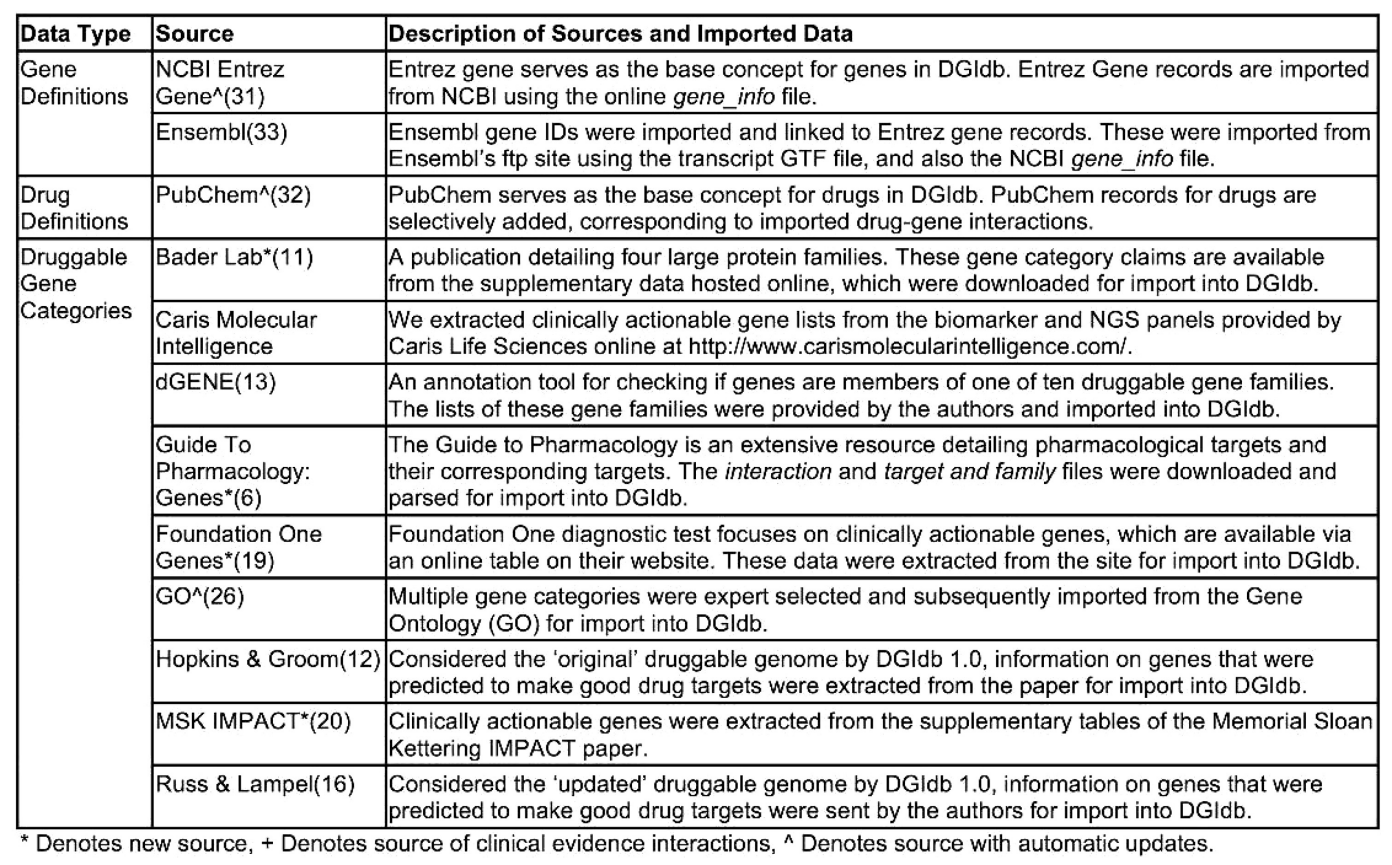		

These additions improve the use of DGIdb as a tool for precision medicine hypothesis generation. Clinical researchers that wish to make sense of lengthy gene lists may use DGIdb to address numerous seemingly simple questions. One natural question is to ask if there is any information on interactions between drugs and their candidate gene list. Alternatively one might ask if any of the genes in that list belong to potentially druggable categories, such as kinases, G-protein coupled receptors or transporters. DGIdb provides an easy-to-use interface for seeking answers to these types of questions. In the process, DGIdb handles the complexity of gene and drug name ambiguity, provides informative results that detail what resources supplied the interaction or druggable gene category, and summarizes key metadata from each resource along with hyperlinks to the original drug and gene concepts (as applicable). This reduces the burden of needing to search and decipher the results of numerous resources (many of which are lists from supplementary tables in papers or other static datastores). Using DGIdb, the clinical researcher may instead focus their attention on reviewing the interesting results of these queries instead of exhaustively searching the numerous resources aggregated and curated by the DGIdb.

## EXPANDED CONTENT

DGIdb 2.0 adds significantly to the existing content, providing a broader view of drug–gene interactions and potentially druggable genes from a large number of sources (Figure 1). Most of this new content (89.5%) comes from sources not present in DGIdb 1.0 and the remainder from those original sources that have been updated with DGIdb 2.0. The top priority for new additions was that they describe high-quality, clinically actionable drug–gene interactions. First among these is the Clinical Interpretation of Variants in Cancer (CIViC) web resource: an open-source, community-driven site for the collaborative curation of clinically actionable variants in cancer. At time of last import, CIViC contained 189 evidence statements from 112 published sources describing genes with variants that predict sensitivity or resistance to specific drugs. In total 218 drug–gene interactions were imported into DGIdb from these evidence statements. A second, similar resource, is the open-source Database of Curated Mutations (DoCM). Unlike CIViC which is limited to mutations with proven clinical relevance, DoCM's scope includes any highly curated variant with a demonstrated role in cancer etiology. A key dataset included in DoCM is the collected annotations of clinically actionable mutations as reported by the Gene Drug Knowledge Database (27).

In addition to CIViC and DoCM, six other notable, curated sources of clinical evidence-based drug–gene interactions were integrated in the DGIdb 2.0 release. Rask-Andersen *et al*. (15) released a carefully curated list of putatively novel clinical trial drug targets, based upon mining CenterWatch's Drugs in Clinical Trials Database (centerwatch.com). Their list of clinical drug–gene interactions is available as a supplementary table in their publication, and was imported into the DGIdb as the ‘Druggable Genome: Clinical Trials’ (‘TdgClinicalTrials’) resource (5,998 claims). My Cancer Genome (MCG) (8) is an original resource included in DGIdb 1.0 (21). However, since the DGIdb 1.0 release, a My Cancer Genome clinical trials search function was introduced. This search tool was used to manually collect information for any clinical trials involving drug–gene interactions. The results were recorded in a table and subsequently imported into DGIdb (1913 claims). Unfortunately, this search functionality is no longer available on the My Cancer Genome site. The Clearity Foundation (clearityfoundation.org) works with patients with ovarian cancer to identify therapeutic strategies guided by biomarkers. To support this effort they have created a database of actionable biomarkers through analysis of thousands of patient tumors. These biomarkers, and their effect on drug sensitivity, constitute drug–gene interactions. With permission from Clearity Foundation, these interactions were extracted from their website and imported into DGIdb (165 claims). In addition, Clearity provided us with 124 manually curated clinical trial records with at least one drug–gene interaction for review and import into DGIdb as the Clearity Foundation Clinical Trial resource (585 claims). DGIdb also includes drug–gene interactions from Cancer Commons (cancercommons.org), a group that formerly enrolled patients with lung cancer, skin cancer, and prostate cancer to profile their tumors and help connect patient tumor profiles with potential therapies, in a self-described ‘macro-scale N-of-1 adaptive trial’. This group curated clinical trial records for genes targeted by FDA approved drugs in these three diseases. The resulting lists of drug–gene interactions, annotated with existing or ongoing collection of clinical data, are available online as HTML tables, and were extracted for import into DGIdb. Finally, DGIdb now includes 1,192 high-quality drug–gene interactions involving FDA approved drugs with single gene targets from the ChEMBL 20 database (28).

The largest source of new content was imported from the IUPHAR/BPS Guide To Pharmacology (GTP) (6), accounting for 10 225 interaction claims and 1,969 druggable gene category claims. GTP includes a detailed arrangement of both genes (targets) and compounds (ligands) into functional categories. GTP also provides groupings of gene targets into families, which we mapped to existing druggable gene categories through an extensive curation effort (Supplementary Methods).

Beyond those added from the GTP, druggable gene category claims were added from four additional sources. The Bader Laboratory (11) druggable gene category claims describe four protein families: Nuclear Hormone Receptors, Kinases, Ion Channels, and Methyltransferases, long considered to be druggable (16). Prior to import into DGIdb, these claims were only available online as supplementary tables (11). The Foundation One (19) diagnostic test focuses on clinically actionable genes, and DGIdb 2.0 uses the data from this source as a new ‘clinically actionable’ druggable gene category. As with the Bader Lab resource, these genes were obtained from an online table on their website (foundationone.com/genelist1.php). The ‘Clinically Actionable’ gene category also had additions from cancer-focused genetic testing panels from Memorial Sloan Kettering (20) and Caris Life Sciences (http://www.carismolecularintelligence.com/profilemenu).

With the content from these new sources, there is an abundance of clinical evidence data to support drug–gene interaction queries. Currently, DGIdb contains 26,298 unique drug–gene interaction claims involving 2,644 genes and 7,569 drugs, and 7,524 unique genes belonging to one or more of 41 potentially druggable gene categories. A total of 8,419 unique genes either have known or potential druggability. We are still far from completely illuminating the ‘dark matter’ of the druggable genome (Isserlin *et al*., http://arxiv.org/abs/1102.0448); of the genes in potentially druggable gene categories, only 26.4% (1,983) actually have a known drug–gene interaction (Supplementary Figure S1). Moreover, it is likely that only a fraction of these represent effective targeted therapies. Conversely, only 75.0% (1,983/2,644) of unique genes with at least one drug–gene interaction belong to an existing druggable gene category. The latter illustrates our potentially still inadequate definition of the ‘druggable genome’, but does represent a modest improvement over the 65.2% (1,704/2,611) coverage observed in DGIdb 1.0.

**Figure 2.**
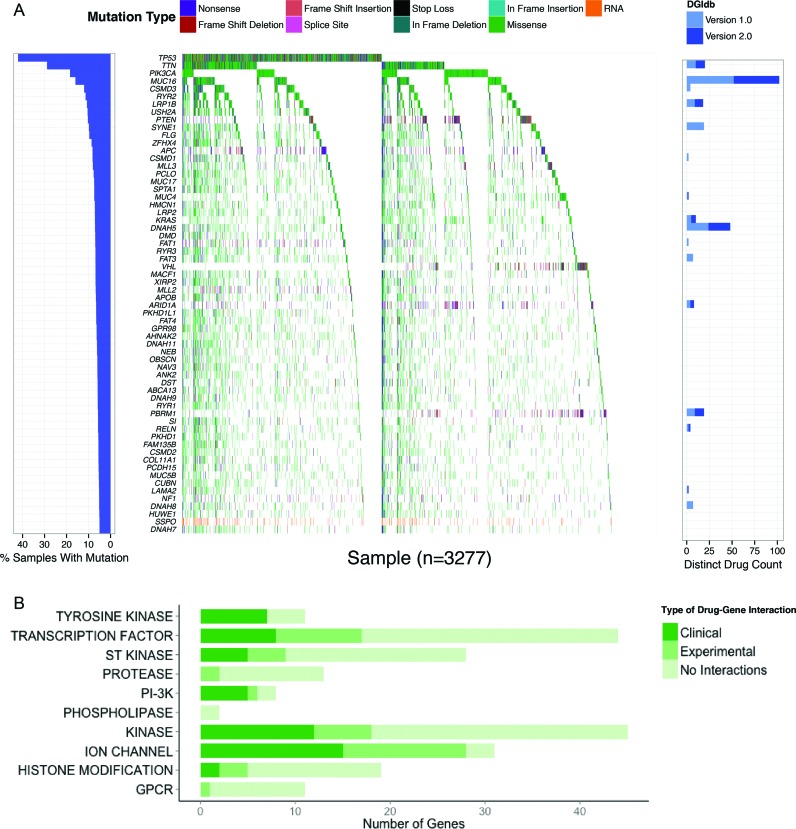
DGIdb analysis of TCGA pan cancer recurrently mutated genes. (**A**) The 62 genes recurrently mutated in at least 5% of pan-cancer tumors, and the corresponding mutations observed in each tumor. (**B**) The number of potentially druggable genes among the 488 genes recurrently mutated in at least 2.5% of tumors, grouped by druggable gene categories. Colored bars indicate the fraction of each such category with interactions in a clinical evidence source, a non-clinical evidence source, or without interactions. GPCR = G-Protein Coupled Receptor, PI-3K = Phosphatidylinositol 3 Kinase, ST Kinase = Serine Threonine Kinase.

To demonstrate an application of the expanded content of DGIdb 2.0, we evaluated pan-cancer data from The Cancer Genome Atlas (TCGA) (29), and used the R package GenVisR (https://github.com/griffithlab/GenVisR) to illustrate the potential druggability of highly recurrent mutated genes in this cohort (Figure 2A). Of the top 62 recurrently mutated genes (those mutated in at least 5% of the evaluated tumors), 16 have known drug–gene interactions in DGIdb 2.0. Moreover, multiple drugs are available for 14 of these. This is a notable improvement from DGIdb 1.0, with nearly a three-fold (193.6%) overall increase in the number of drug–gene interactions for this set. A search of the top 488 recurrently mutated genes (those genes mutated in at least 2.5% of the evaluated tumors) shows that 137 genes (28%) are present in at least one potentially druggable gene category (Figure 2B). Of these, 57 genes (42%) have known drug–gene interactions, and 30 genes (22%) have drug–gene interactions that are based on data describing clinical evidence. These results demonstrate the usefulness of DGIdb as a tool to explore potential hypotheses for clinically actionable drug interactions with implicated cancer genes.

## NEW FUNCTIONALITY

**Figure 3.**
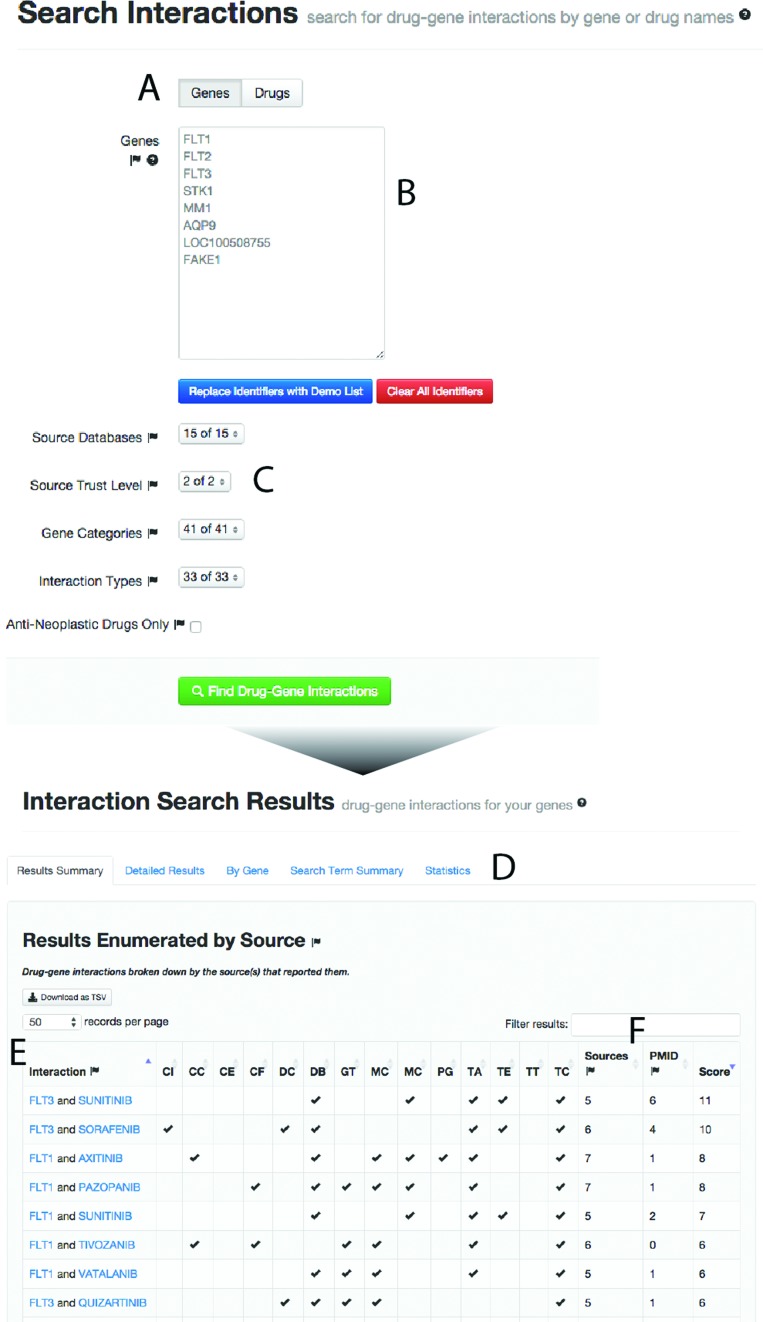
The drug search interface. (**A**) Switching between gene and drug searches is as simple as selecting the mode with this button. (**B**) A search field that accepts a variety of drug or gene identifiers, and provides autocompletion suggestions for terms in the database. (**C**) Filters allow search results to be limited by the source database, curation level, and interaction type. (**D**) Users may select to review the results summary or more detailed views. (**E**) The results summary links genes and drugs by interaction and shows which of the queried resources contain interaction data. (**F**) The unique sources and PMIDs associated with each interaction are displayed to the user. These are combined to provide a score value that may be used for ranking the interactions.

Drug-based search is a new and often-requested feature introduced in DGIdb 2.0, and is a major improvement to the existing search capabilities. DGIdb 1.0 provided a search form for entering mixed gene identifiers (IDs) including gene symbols, Entrez IDs, UniProt symbols, etc., to get a list of drug–gene interactions (Figure 3A). We have expanded the functionality of this form to alternatively use drug IDs for the search process (Figure 3B). These may take the form of any of the varied IDs in the database, including PubChem Compound and Substance IDs, Drugbank IDs, common drug names, brand names, CAS numbers, etc. The drug search results work in much the same way as the gene search results (21). Drug terms are first matched against PubChem primary names, then against any other identifiers. If an exact match is found to a DGIdb drug, DGIdb searches for drug–gene interactions involving that drug. Filters allow the search space to be constrained by source, interaction type, trust level or gene category (Figure 3C). If any search terms are ambiguous (i.e. the search term matches more than one drug concept) or no matches are found, this information is summarized in the ‘Search Term Summary’ tab and in detail in the ‘Detailed Results’ tab (Figure 3D). On the default ‘Search Term Summary’ tab, interactions are grouped together and the sources supporting each interaction are indicated (Figure 3E). Finally, an indication of the number of distinct sources and distinct PubMed IDs (PMIDs) supporting each interaction are provided, along with a score that accounts for both of these values. The results are sortable by inclusion in any particular database or by the described metrics (Figure 3F).

Finally, DGIdb 2.0 now features a codebase supporting the automatic update from sources for each of the core concepts in DGIdb: genes, drugs, drug–gene interactions and druggable gene categories. These updaters have been built for the most actively updated existing sources (Figure 1), including DrugBank (10) (drug–gene interactions), Gene Ontology (26) (druggable gene categories), Entrez (30) (genes), and PubChem (31) (drugs). For sources updated from version 1.0 to 2.0, the smallest increase we observed was for PubChem, at 1,023 additional drugs (10%). The largest increase was for Entrez Gene, which increased by 13,348 genes (31%), and precisely reflects the corresponding increase in gene identifiers as reported by Entrez (Supplementary Figure S2). These large changes highlight the importance of a continually updated resource, particularly for resources that are applicable to precision medicine hypothesis generation.

## CONCLUSION AND FUTURE DIRECTIONS

DGIdb 2.0 has greatly expanded in content for both drug–gene interactions and druggable gene categories. The numerous sources describing clinical evidence of drug–gene interactions have created a useful resource for reviewing the nature of these interactions in a clinical setting. These additions have been made with the express purpose of improving DGIdb for use in clinically relevant applications. DGIdb remains a powerful tool for web-based searches, and also as an add-on service for clinical informatics pipelines through the well-documented API. This aspect of DGIdb has been improved with the addition of drug-based searches of drug–gene interactions, enhancing the facility with which DGIdb can mine the druggable genome. Automated content updates for large, regularly updated sources now ensures that DGIdb 2.0 will provide users with the most current knowledge of clinically actionable drug–gene interactions.

As DGIdb continues to grow, we anticipate additional improvements to accommodate the complexity of representing drug–gene interactions and druggable gene category assignments. A future goal is to better represent many-to-one relationships between genes and drugs for interactions. This would include both multi-drug interactions with individual gene targets, and drugs that simultaneously interact with multiple genes. These types of data will become increasingly available for aggregation and representation as clinical data describing these events are entered as evidence in resources such as CIViC. We also plan a corresponding expansion of our API, adding options for queries that take advantage of these new data relationships. We also wish to study a more sophisticated scoring and ranking system that takes into account other metrics, such as source quality and FDA drug information. Additionally, we plan to investigate better strategies for the grouping of related drug concepts. Finally, we plan to continue adding new sources of drug–gene interactions as identified by our group or suggested by users of the resource. Several features and sources of the DGIdb were developed through feedback from this community, for which we express our gratitude.
